# Supplementary material for: The reciprocal relation between morphological awareness and spelling in Chinese: A longitudinal study of primary school students
Source: PLoS One. 2020 Dec 21;15(12):e0243050. doi: 10.1371/journal.pone.0243050 (PMC7751876; doi:10.1371/journal.pone.0243050)
Supplement: Attachment — Submitted filename: Response_to_Reviewers.docx [file pone.0243050.s002.docx]

**Author’s Responses to Reviewer Comments**

Dear Editor,

We appreciate the comprehensive and thoughtful reviews of our manuscript titled, “*The reciprocal relation between morphological awareness and spelling in Chinese: A longitudinal study of primary school students* *(PONE-D-20-01004)*,” which we are now resubmitting to *PLOS ONE* for consideration. The reviewer comments were very helpful and we have revised the manuscript accordingly. In particular, based on the reviewers’ comments, we revised the introduction and discussion sections, and added the “the Theory of Spelling Development” to support this research. Subsequently, the content of related tests in this study was also refined. The main corrections in the paper and the responds to the reviewer’s comments are as follows:

**Reviewer 1:**

This is a very strong manuscript that shows potential to be of great interest to a broad audience of researchers - both in the fields of reading and spelling development and those interested in the applicability of models that had been predominantly developed in alphabetic languages (such as English) to other languages (reading and spelling research has been dominated by models based on English speakers, yet it is important to disentangle linguistic influences from cognitive processes). This is a well-designed study, and the findings will move the field further. However, there are a couple of recommendations that may enhance the quality of this manuscript:

**Reviewer’s comment 1.** The relevance of this study could be augmented by linking it to universals in reading development. Chen & Pasquarella have a chapter about reading acquisition in Chinese in Verhoeven and Perfetti’s (2017) book, Learning to read across languages and writing systems. This chapter could be helpful in situating this study in the broader field, so its impact can be enhanced.

***Author’s Responses:*** *Thank you for your comments. According to your suggestions, we have adopted Verhoeven and Perfetti’s study to augment the relevance of this study by linking it to universals in reading development. We added “The influence of morphological awareness on spelling may be common across languages in the writing system [7,11,17]. Even though Chinese characters are a special ideograms, many empirical studies [1,4,6,11] have found that morphological awareness contributes significantly to reading and spelling.” on the page 6 in the* **Introduction section***.*

*Verhoeven L, Perfetti C. (Eds.). Learning to read across languages and writing systems. Cambridge, UK: Cambridge University Press; 2017.*

**Reviewer’s comment 2.** It would be helpful to provide motivation for the longitudinal aspect of the study (the design itself is a strength). This may be accomplished by discussing models of spelling development. That may also help with the finding that morphological awareness and spelling share a bidirectional relationship in grades 1 and 2, but unidirectional to grade 3.

***Author’s Responses:*** *We really appreciate your advice. We have added the “Spelling Development Theory” in the* **Introduction section***. For example: “The Theory of Spelling Development [23] suggests that the development of spelling is a series of developmental processes. Brown [23] recognizes that children in the first and second grades acquire basic spelling skills, such as specific letters representing specific sounds, which may involve phonetic strategies. Subsequently, children in the third and fourth grades are familiar with suffixes, compound words, and homophones. This stage involves more strategies for shape and semantics. Shen and Bear [24] suggest that similar to English children, Chinese children’s spelling follows the same development stage. They then found that as the grade increases, children would use morphemes and semantic strategies more frequently. In response to the previous studies, morphological awareness plays an important role in the development of spelling.”*

*We have added the statement of “From the perspective of spelling development, although morphological awareness and spelling are closely related, it is unclear whether there is a bidirectional relationship between morphological awareness and spelling longitudinally over time.” on page 8 in the* **Introduction section***.*

*As shown in comment 6, we also explained the results of this study from the stage of spelling development in the* **Discussion section.**

*Shen HH, Bear DR. Development of orthographic skills in Chinese children. Reading and Writing. 2000; 13(3), 197－236.*

*Brown A. A review of recent research on spelling. Educational Psychology Review. 1990; 2, 365–397.*

**Reviewer’s comment 3:** In the method section, it would be helpful to have a little more information about the instructional context. For example, how many years of instruction preceded first grade? What was reading instruction like (e.g., was pinyin included? was a look-say method used?).

***Author’s Responses:*** *Thank you for requesting more details about the instructional context.* *We have added “In Mainland China, children go through three years of kindergarten education before entering primary school. Formal literacy instruction is not given in kindergarten, which begins in the first grade of elementary school. When students enter the first grade, they receive at least two months of pinyin training. Subsequently, teachers instruct students by spelling Chinese characters with pinyin on the blackboard using stroke-by-stroke. At the same time, students are required to imitate spelling.” on page 9 in the* **Method section.**

**Reviewer’s comment 4:** It would be helpful to have a little more information about the measures. What were the maximum scores? Were they used and validated in past research? The description of the measures themselves were also a little challenging to follow.

***Author’s Responses:*** *Thank you very much for the suggestion. We have added the maximum scores about homograph awareness, homophone awareness, compounding awareness and spelling. For example: The statement “the maximum score was 24” has been added to homograph awareness test. The statement “There were no maximum scores for this task.” has been added to homophone awareness test. The statement “The maximum score was 60” has been added to compounding awareness test and the statement of “The maximum score was 36” has been added to spelling.*

*In method section, we used many previous tests. Homophone awareness, homograph awareness, compounding awareness, phonological awareness and orthographic awareness were adopted from Li and Wu’s study (2015), in which the Cronbach’s α coefficient of the three tests was 0.90, 0.88, 0.90, 0.71, and 0.79, respectively. Spelling test was adopted from Li, Wu, Cheng, and Nguyen’s study (2019), in which Cronbach’s α coefficient was 0.75.*

*The Cronbach’s α coefficient of this study has been presented in a table, as shown in the table below. Therefore, tests are* validated*. Furthermore,* *these coefficients are also presented in the manuscript, as shown in table 1.*

| variables | Cronbach’s α coefficient | | | | | |
| --- | --- | --- | --- | --- | --- | --- |
|  | | Time 1 |  | Time 2 |  | Time 3 |
| IQ | | 0.93 |  |  |  |  |
| Phonological Awareness | | 0.85 |  |  |  |  |
| Orthographic Awareness | | 0.79 |  |  |  |  |
| Homophone Awareness | | 0.90 |  | 0.72 |  | 0.82 |
| Homograph Awareness | | 0.87 |  | 0.78 |  | 0.70 |
| Compounding Awareness | | 0.88 |  | 0.80 |  | 0.78 |
| Spelling | | 0.75 |  | 0.74 |  | 0.75 |

**Reviewer’s comment 5:** The tables would be easier to follow with the variable names written out in full.

***Author’s Responses:*** *We are very grateful for your suggestion. According to the reviewer’s suggestions, we have written out the variable names in full in table 1, table 2 and* *Figure 1.*

**Reviewer’s comment 6:** The interpretation of the findings would be strengthened if they were connected to broader models of spelling development and universals. This is a strong study, in and of itself. However, it would benefit from being connected to broader models of learning to spell and how the linguistic features of Chinese may lead to different cognitive patterns of performance. ***Author’s Responses:*** *Thank you for your insightful comments. As discussed in comment 2,* *to explain the findings of this study, we also added the Theory of Spelling Development in the* **Discussion section***. For example, “According to the Theory of Spelling Development [23], it is a critical period of spelling development from first grade to second grade. Children must master a large number of Chinese characters and have had extensive exposure to print at this stage. Thereafter, frequent spelling experience and some formal guidance can enable children to acquire abstract awareness, i.e., morphological awareness. Morphology and semantic strategies become increasingly important to children as they grow older. Thus, spelling skills facilitate the subsequent development of morphological awareness.” on page 18. Your meaningful suggestion has inspired us a lot. Please see these clarifications in the* **Discussion section.**

**Reviewer 2:**

**Reviewer’s comment 1:** I believe that this study could makes a meaningful contribution to the literature on the relationship between spelling and morphological awareness in Chinese. However, some revision is necessary before it is ready for publication. There are some parts that I do not understand well enough to be able to meaningfully evaluate whether the conclusions drawn are supported by the data. The manuscript should be revised in collaboration with a native speaker of English familiar with the topic of the paper. There are some cases where it is difficult for me to tell whether the problem is one of theoretical imprecision or simply a matter of wording. My primary concerns about this paper have to do with the measures.

***Author’s Responses:*** *Thank you very much for your comments.* *After careful revision*, *we submitted the revised manuscript to* *a language-editing service company Editage (www.editage.cn), for English language editing, to help improve the grammar, syntax, and flow of our manuscript. The related certificate is uploaded as a separate file.*

**Reviewer’s comment 2:** The homophone task (page 9) is described in two ways that seem to contradict each other: In lines 175-177 the description is as follows: “This test would assess children’s ability to manipulate the homophone morphemes which owned the same pronunciation and the different meaning and grapheme,” This makes it sound like the child is being asked to provide words with containing a different but homophonous character. The example given later in the paragraph is consistent with this description. However, the following lines (177-179) say: “The experimenter orally presented the target morphemes in a compound word, then asked children to produce other words using the target morpheme.” The phrase “the target morpheme” would seem to seem to imply that the child is supposed to produce words with the same morpheme/character. This may be an imprecise use of the phrase “target morpheme” but such an error should not occur in a paper on morphology. Furthermore, it is not specified how a response would be scored if the child produced a word using the same character and meaning, or a word using the same character with a different meaning (in this case, “ocean'). If a correct response requires the child to produce a word with a different character – i.e., a different spelling – then this test, though administered orally, is actually to a substantial degree a spelling test.

***Author’s Responses:*** *We apologize for not expressing the homophone task clearly, which caused this misunderstanding. We have revised this task according to the reviewer’s comments. We have modified the homophone awareness test, for example: “The experimenter orally presented the target morphemes in a compound word, such as the target morpheme /yang2/羊(sheep) in /shan1yang2/山羊(sheep). Children were asked orally to produce one word (e.g., /yang2rou4/羊肉mutton) containing the same meaning as the target morpheme to let children clearly understand what the target morpheme is, but this word (e.g., /yang2rou4/羊肉mutton) is not scored. Thereafter, the children were asked orally to produce another word (e.g., /tai4yang2/太sun) containing a different meaning as the target morpheme and was scored one point. The experimenters encouraged children to produce more homophone words with a different meaning. The more homophone words children produced, the higher the score. There were no maximum scores for this task.” Please see these clarifications in the* **Method section***.*

*The experimenter presented the homophone awareness task orally, and the children were asked* *orally to express the homophone words with the different meaning. This task has nothing to do with graphemes and spelling systems. Therefore, this task is different from spelling. We would thank you again for your advice.*

**Reviewer’s comment 3:** Likewise, from the instructions for the homograph test, it appears that this test requires the child to produce a word that is written with the same character, but a distinctly different meaning. It is not specified how a response would be scored if the child produced a word with a different, homophonous character. If in fact to be correct the child must produce a word which contains the same character, but with a different meaning, this too is at least in part a spelling test.

***Author’s Responses:*** *Thank you very much for your suggestion. Children were required* *to produce two words in the homograph awareness test:* *one has the same shape and the same meaning as the target morpheme, another word has the same shape and different meaning as the target morpheme. Both words were scored. We have added this statement on page 11, “/mian4tiao2/面条(noodle) was scored one point. /mian4rong2/面容(face) was also scored one point.”*

*Like the comment in the second question, “the experimenter presented the homograph awareness task orally, and the children were asked orally to express two words.” This task has nothing to do with graphemes and spelling systems. Therefore, this task is different from spelling.*

**Reviewer’s comment 4:**About the spelling test (pages 10-11): I can imagine different types of spelling errors in Chinese, that would relate to morphological awareness in different ways. For example, a child might spell a word with the wrong but homophonous character (one with the same sound, but a different meaning). And in this case, there is a further distinction between homophonous characters sharing the same phonetic component, and homophonous characters with nothing in common. Another type of error might be giving a character graphically similar to the correct one. A third type of error might be missing or misplacing a stroke in a character. Which types of errors actually occurred in the data? Might a more nuanced scoring system, distinguishing among error types, lead to more insight?

***Author’s Responses:*** *Thank you for your thoughtful suggestions. Just as you suggested, the previous study (Shen & Bear, 2000) has found three spelling errors, such as orthographic errors, phonological errors, and semantic (morpho-lexical) errors in the spelling of Chinese characters. Analyzing error types could lead to more insight. However, our study focuses on the bidirectional relationship between morphological awareness and spelling, so spelling error types are not presented in this study. We also think that error types are a meaningful and interesting field that it can be discussed in a future study.*

*Furthermore, we have added* *“On the other hand, this study only investigates the correct spelling of Chinese characters, and the errors types were not analyzed. Investigating spelling error types foster a foundation for the improvement of spelling ability and the development of orthographic awareness. Therefore, it should be considered as the meaningful and interesting field that can be discussed in a future study.”* *in the* **Implications and future research section*.***

*Shen HH, Bear DR. Development of orthographic skills in Chinese children. Reading and Writing. 2000; 13(3), 197－236.*

**Reviewer’s comment 5:** I did not clearly understand the explanation of why there was not an effect of spelling on later morphological awareness after grade 1.

***Author’s Responses:*** *Thank you very much for your suggestion. We have carefully revised the manuscript. For example: “One possible reason is the change of the spelling development stage. The Theory of Spelling Development indicates that children already have some spelling experience from the first stage to the second stage. Chinese children have mastered about 1,300 Chinese characters. Spelling experience could help children perceive orthographical rules. During the second spelling stage, children have developed a higher level of orthography, which is close to the adult level in Chinese [30]. Children who are more familiar with spelling rules and spelling action achieve to the level of automation. The individual differences in spelling become smaller, leading to the explanation of less variance of morphological awareness. Therefore, spelling may not contribute to the morphological awareness.*

*Another possible reason is the difference of spelling development rate. The spelling development rate is fast in the first grade, but it slows down in the second grade because children are required to manipulate the higher level cognitive tasks, such as writing structure and thinking [31]. The development of morphological awareness would need a broader context of reading-related skills. A slower spelling development does not provide a broader knowledge background. Children may gain more cognitive resources and linguistic skill by reading than spelling in second stage. Therefore, reading may play a more important role in the development of morphological awareness. Previous study [25] has also found that reading affects the development of children’s morphological awareness. The development of morphological awareness could be more affected by reading than by spelling. Hence, the subsequent morphological awareness was not significantly affected by the slower spelling development. The current study found that there is the unidirectional relationship between morphological awareness and spelling from the second to third grade, and the unidirectional relationship might be unique in Chinese. This conclusion also needs to be confirmed with further studies in other languages.”* *in the* **Discussion section***.*

We have satisfactorily addressed all criticisms and concerns raised by the reviewer and hope that you will accept our revised manuscript for publication in *PLOS ONE.* We look forward to hearing from you soon.

Yours sincerely,

Authors
